# Parallel colonization of subalpine habitats in the central European mountains by *Primula elatior*

**DOI:** 10.1038/s41598-019-39669-2

**Published:** 2019-03-01

**Authors:** Veronika Konečná, Michael D. Nowak, Filip Kolář

**Affiliations:** 1 Department of Botany, Faculty of Science, Charles University, Benátská 2, CZ-128 00 Prague, Czech Republic; 2 Institute of Botany, The Czech Academy of Sciences, Zámek 1, CZ-252 43 Průhonice, Czech Republic; 3 Natural History Museum, University of Oslo, Sars’ gate 1, NO-0562 Oslo, Norway; 4 Department of Botany, University of Innsbruck, Sternwartestraße 15, AT-6020 Innsbruck, Austria

## Abstract

The island-like distribution of subalpine habitats across mountain ranges can trigger the parallel evolution of locally adapted ecotypes. Such naturally replicated scenarios allow testing hypotheses on how elevational differentiation structures genetic diversity within species. Nevertheless, the parallel colonization of subalpine habitats across different mountain ranges has only rarely been documented with molecular data. We chose *Primula elatior* (Primulaceae), naturally spanning entire elevation range in multiple mountain regions of central Europe, to test for the origin of its scattered subalpine populations. Nuclear microsatellite variation revealed three genetic groups corresponding with the distinct study regions. We found that genetic differentiation between foothill and subalpine populations within each region was relatively low, suggesting that the colonization of subalpine habitats occurred independently within each mountain range. Furthermore, the strongest differentiation was usually found between the subalpine populations suggesting that mountain ridges may act as migration barriers that can reduce gene flow more strongly than elevational differences between foothill and subalpine populations. Finally, we found that subalpine colonization did not result in a loss of genetic diversity relative to foothill populations in agreement with the high migration rates that we document here between the subalpine and the foothill populations. In summary, our study shows subalpine *Primula elatior* populations are genetically diverse and distinct results of parallel colonization events from multiple foothill gene pools.

## Introduction

Alpine and subalpine habitats represent challenging and often unpredictable environments for plants. Plants have to face a complex set of stresses in such environments including freezing, fluctuating temperatures, increased UV radiation, and terrain disturbances implying various selection pressures^1^. Local adaptation to these environmental stresses often results in the evolution of specific morphological and/or physiological traits that confer increased fitness in these challenging environments^2,3^. Populations characterized by these adaptations are often called “ecotypes” and their level of overall genetic differentiation from ancestral populations may be still very low^2,4^. In cases where reproductive barriers arise between ecotypes and their ancestral populations, either as a consequence of natural selection or simply due to spatial isolation, the ecotype may represent the first step towards the founding of a new species^5,6^. In such cases, we can observe speciation as a continuum of divergence along an elevational gradient leading to textbook examples of ecotypic differentiation^7,8^. The restriction of gene flow between lower and higher elevations as well as the reduction or absence of gene flow among mountain ridges can lead to the accumulation of reproductive barriers and/or hybrid incompatibilities^9^. This process is but one of several potential explanations for relatively high species diversity in alpine/subalpine habitats throughout the world^10^.

The island-like distribution of alpine/subalpine habitats can trigger allopatric differentiation and parallel colonization from lower elevations independently in each mountain range^11^. It can also lead to phenotypic differentiation potentially resulting in the convergent evolution of traits that confer local adaptation (i.e. ecotypes)^2^. Although the mountains provide an ideal model system for studying the evolution of recurrently adapted ecotypes, the parallel colonization of alpine/subalpine habitats has only been rarely documented with molecular data. Rare exceptions to this are represented by studies in *Arabidopsis halleri*^12^, *Zea mays*^13^, and *Populus trichocarpa*^14^. The alternative scenario of a single origin of alpine/subalpine ecotype followed by dispersal has also been documented, for example in *Senecio halleri* whose alpine populations represent a single lineage that colonized the Alps in a stepwise manner^15^.

The influence of habitat differentiation on the genetic diversity of populations has been previously studied mainly at lower elevations^16–18^. It is poorly known how the colonization of alpine/subalpine h abitats has shaped the genetic diversity of populations, but it is hypothesized that alpine/subalpine populations would be genetically depauperate relative to lowland populations due to the effects of genetic bottleneck, genetic drift, reduced gene flow, and habitat fragmentation^19^, all of which can promote the genetic isolation of alpine/subalpine populations from their foothill relatives^20^. This has indeed been observed in alpine populations of *Arabidopsis thaliana* from the Italian Alps, which exhibit reduced genetic diversity relative to foothill populations^21^, but in contrast, alpine *A*. *thaliana* populations from the Swiss Alps show no evidence of reduced genetic diversity^22^. In *Primula merrilliana* from eastern China, alpine populations exhibit even higher genetic diversity, moreover, they are larger and more inter-connected with gene flow than foothill populations^23^. This pattern implies that the foothill populations may have been colonized by alpine populations. In summary, studies of recurrently originated ecotypes of a single species may provide valuable replicates to test the generality of how alpine conditions shape population genetic diversity.

In this study, we address the genetic consequences of elevational differentiation in *Primula elatior* (Primulaceae), a species with broad ecological preferences including a large elevational range in several mountain ranges in Europe^24^. The wide ecological breadth is linked to high morphological variation primarily in leaf and calyx shape^24,25^. Individuals originally from subalpine populations tended to have urceolate calyxes (the narrowest in the top part of calyx), compared to individuals from foothill populations mainly with tubular calyxes (the same wide along the calyx)^26^. The morphological traits are plastic, except the shape of calyx, which remained stable after cultivation of subalpine population under uniform foothill conditions in a common garden experiment in one of the mountain ranges (the Krkonoše mountain range, V. Konečná unpubl.). In contrast to morphological investigations, the genetic structure of *P*. *elatior* remains unknown except studies at fine-scale in Belgium^16,27–29^. We focus on three mountain ranges in central Europe, where *P*. *elatior* grows along an elevational gradient from foothill meadows, river banks, and forest edges, up to subalpine meadows, snowbeds, and rocky outcrops in the glacial cirques. In the Krkonoše and the Jeseníky mountains, subalpine populations are restricted to glacial cirques. In the Tatry mountains, subalpine populations grow in valley meadows and snowbeds. Generally, the difference between foothill and subalpine populations across mountain ranges is defined by treeline, foothill populations occur below the treeline in contrast to subalpine populations, which occur above the treeline. We assume colonization of subalpine habitats from foothill habitats during warmer periods of the postglacial Holocene. Our assumption of upslope colonization is likely because temperate species are highly unlikely to have survived past glaciations that affected these subalpine habitats^30^.

Using *P*. *elatior* as a suitable system, we examined how elevation shapes genetic structure within a species, and by comparing these results across three distinct mountain ranges (the Jeseníky, the Krkonoše, and the Tatry), we evaluated how generally applicable these patterns of differentiation might be in *P*. *elatior*. First, we tested whether the subalpine populations in three mountain ranges represent parallel colonization events of subalpine habitats occurring independently in each mountain range or if the subalpine ecotype evolved once and later spread across the different mountain ranges. Second, we tested if the elevation acts as a barrier to gene flow between foothill and subalpine populations, and whether gene flow is asymmetric; e.g. are migration events from high to low elevations following the downslope transport of seeds and pollen more common than upslope migration events. Finally, we tested for a reduction of genetic diversity associated with the colonization of subalpine habitats relative to lowland habitats.

## Results

### Genetic structure and evolutionary relationships among populations

By genotyping 12 nuclear microsatellite loci in 202 individuals from 16 populations we detected a total of 120 alleles with maximum of 24 and minimum of three alleles per locus.

**Figure 1:**
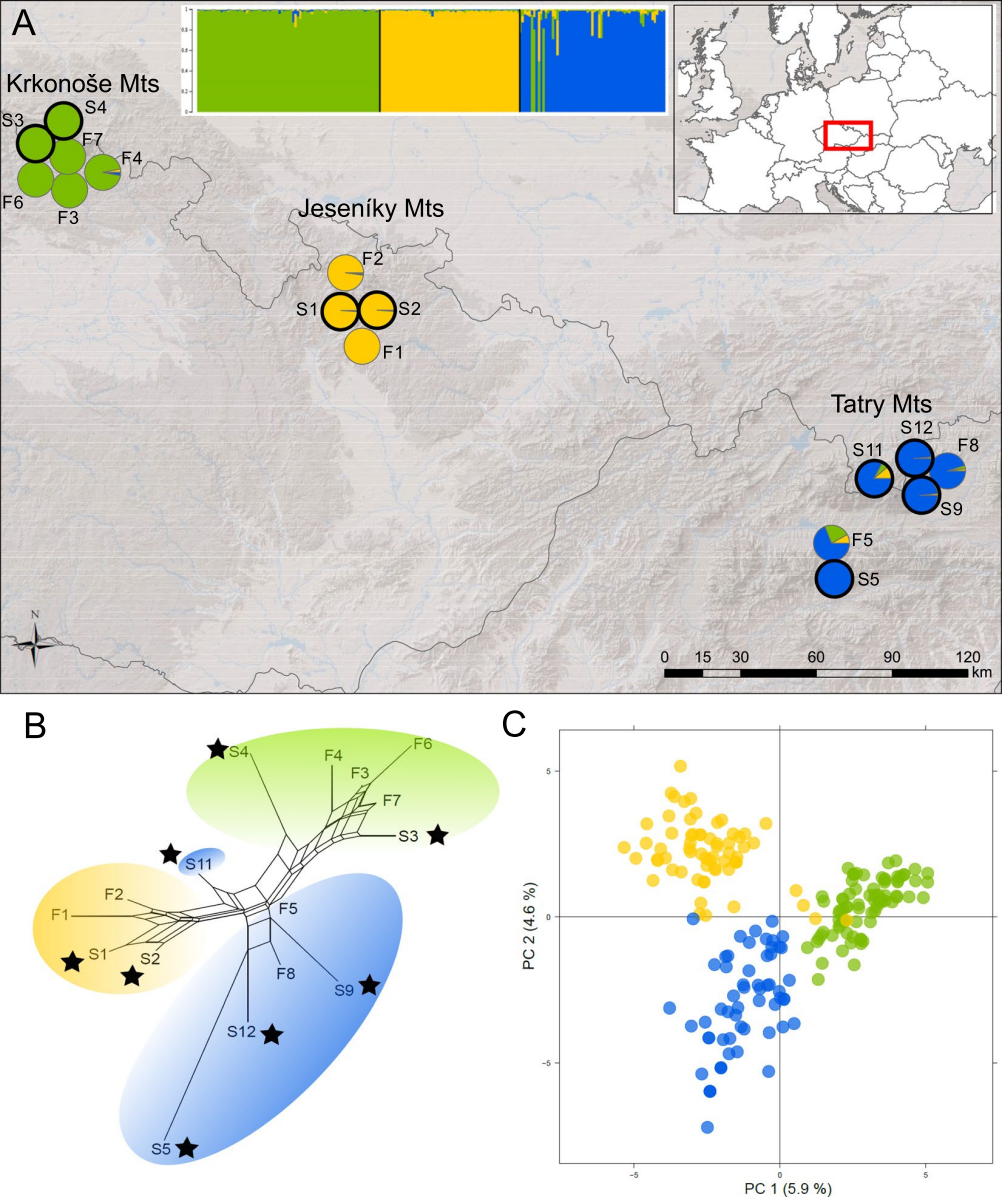
Parallel origin of subalpine populations of *Primula elatior* in the three mountain ranges in central Europe. (**A**) Geographical distribution of the complete dataset of 16 foothill and subalpine (marked by bold circles) populations of *P*. *elatior*, pie charts and barplots show the proportional assignment of individuals from each population to the three clusters inferred by structure, (**B**) neighbor-joining network of the same populations based on among-population F_ST_ distances (subalpine populations marked by asterisks), (**C**) principal component analysis (PCA) of all 202 individuals (coloured according to structure results). Map was processed in ArcMap version 10.0 (http://desktop.arcgis.com/en/arcmap/) by V. Konečná, map layer was modified (http://desktop.arcgis.com/en/arcmap/10.3/manage-data/raster-and-images/hillshade-function.htm) by V. Konečná.

We explored the genetic structure of *P*. *elatior* populations across the three target mountain ranges, where it occupies both foothill and subalpine habitats, using Bayesian clustering (structure), distance networks (Neighbor-joining networks), and ordinations (principal component analysis, PCA). The results of the structure analyses showed that populations from each mountain range (the Jeseníky, the Krkonoše, and the Tatry) formed a separate cluster, regardless of their foothill-subalpine differentiation, under the corresponding partition of K = 3 (Fig. 1A). The model of K = 3 also exhibited the highest similarity among the replicated runs and at this partition the rise of likelihood values started to flatten, suggesting that the inclusion of additional parameters do not significantly improve the fit of the model beyond K = 3 (Supplementary Fig. S1). We observed three clusters according to the three mountain ranges in PCA (Fig. 1C) as well as in Neighbor-joining network based on F_ST_ distances (Fig. 1B). The separation of populations into three clusters that largely corresponded to geographical range and the absence of any genetic structure associated with elevation strongly suggested the parallel evolution of the subalpine populations in each mountain range.

**Figure 2:**
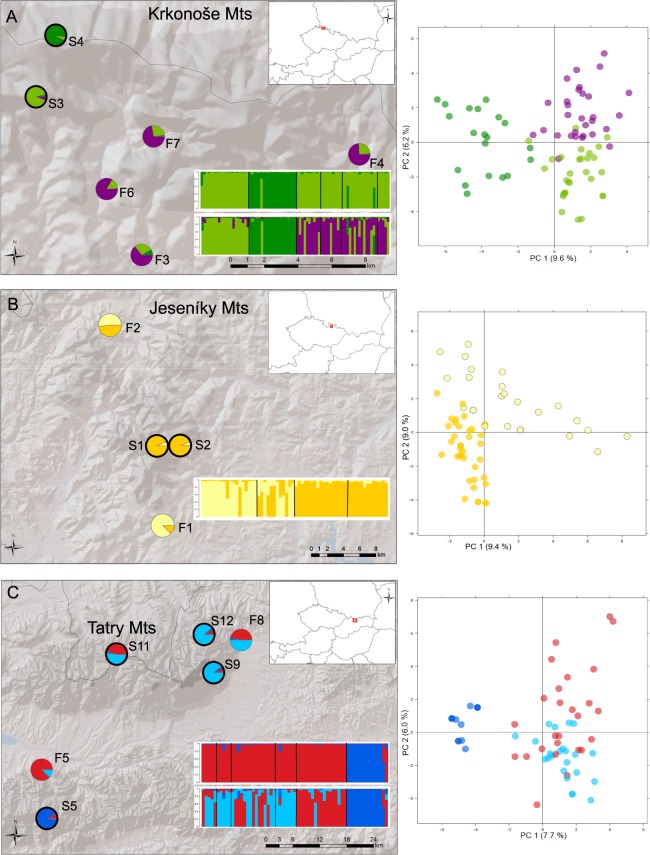
Genetic sub-structuring of *P*. *elatior* populations in each target mountain range. Pie charts and barplots show the proportional assignment of individuals from each population to clusters inferred by a separate structure analysis of (**A**) the Krkonoše (79 individuals), (**B**) the Jeseníky (60 individuals), and (**C**) the Tatry (63 individuals); accompanied by principal component analysis (PCA) of individuals. Subalpine populations are denoted by bold circles. Maps were processed in ArcMap version 10.0 (http://desktop.arcgis.com/en/arcmap/) by V. Konečná, map layers were modified (http://desktop.arcgis.com/en/arcmap/10.3/manage-data/raster-and-images/hillshade-function.htm) by V. Konečná.

In subsequent analyses, we performed separate structure analyses for each region to investigate finer structure within each mountain range (Fig. 2). All the analyses tended to separate the subalpine and foothill populations within each region: this was apparent already under K = 2 in the Jeseníky and under K = 3 in the other two mountain ranges. In the Krkonoše and the Tatry, one subalpine population (S4 and S5, respectively) separated from the remaining populations under K = 2, suggesting that not only one group of subalpine populations exists in these mountain ranges.

### Genetic diversity of populations and among population variation

The populations varied considerably in terms of within population variation. Allelic richness ranged from 2.29 to 3.72 with the highest values (3.33–3.72) being present in foothill populations in the Tatry (Table 1, Supplementary Fig. S2). Similarly, expected heterozygosity (H_E_), ranged from 0.4 to 0.6 in the whole dataset and foothill populations from the Tatry exhibited the highest values (0.52–0.6). Populations also varied in gene diversity (H_S_) ranging from 0.43 to 0.65 with the highest values (0.65–0.61) in one foothill and two subalpine populations from the Tatry. The proportion of rare alleles (DW index)^31^ exhibited a similar trend as well, with the range from 82.44 in the Jeseníky subalpine population to 411.79 in the Tatry foothill population. Despite this variation, we did not detect consistent differences between the group of foothill and subalpine populations across three mountain ranges in diversity index (non-significant ANOVA with region as a random factor) (Table 2). Instead, each mountain range tended to show a different pattern (Supplementary Fig. S2).

**Table 1:** Genetic diversity of 16 populations of *Primula elatior* from foothill and subalpine habitats in the three mountain ranges investigated.

Pop. ID	Locality	Range	Group	Elevation	No. Ind.	No. Alleles	Allelic richness	H_O_	H_E_	H_S_	DW index	No. private alleles	HWE test^a^	MIGRATE-N
F1	Žďárský potok u Rýmařova	Jeseníky	foothill	735	18	41	2.6	0.43 ± 0.28	0.44 ± 0.26	0.43	240.76	2		✓
F2	V Mlýnkách	Jeseníky	foothill	618	13	44	2.71	0.35 ± 0.29	0.46 ± 0.28	0.45	121.95	1	PRIV 4^**^, PACA 78^**^, PV 4767^*^, PV 23741^*^	✓
S1	Velká Kotlina	Jeseníky	subalpine	1397	13	36	2.61	0.49 ± 0.36	0.46 ± 0.30	0.54	82.44	0	PACA 78^*^, PV 23741^*^	✓
S2	Velká Kotlina edge	Jeseníky	subalpine	1301	16	39	2.6	0.46 ± 0.30	0.48 ± 0.27	0.51	84.78	0	PRIV 4^*^, PACA 78^*^	✓
F3	Strážné	Krkonoše	foothill	776	15	45	2.75	0.41 ± 0.37	0.46 ± 0.33	0.49	175.15	2	PRIV 4^***^, PACA 38^**^	✓
F4	Spálený mlýn	Krkonoše	foothill	788	10	39	2.68	0.41 ± 0.35	0.46 ± 0.30	0.49	84.45	0	PACA 38^*^	✓
F6	Michlův mlýn	Krkonoše	foothill	658	9	35	2.52	0.39 ± 0.29	0.43 ± 0.26	0.45	150.71	1	PACA 38^*^	
F7	Svatý Petr	Krkonoše	foothill	816	5	34	2.82	0.43	0.5	0.57	138.8	0		
S3	Velká Kotelní jáma	Krkonoše	subalpine	1280	20	45	2.62	0.40 ± 0.32	0.45 ± 0.31	0.53	145.06	4	PRIV 4^***^, PACA 78^**^	✓
S4	Malá Sněžná jáma	Krkonoše	subalpine	1368	20	41	2.52	0.34 ± 0.32	0.44 ± 0.32	0.52	227.05	1	PRIV 4^***^, PACA 78^**^, PV 4767^*^, PACA 38^*^	✓
F5	Poludnica	Tatry	foothill	1055	17	68	3.72	0.57 ± 0.33	0.62 ± 0.29	0.61	411.79	9	PRIV 4^***^, PV 1973^**^	✓
F8	Ždiar, Tatranská Kotlina	Tatry	foothill	769	15	63	3.33	0.48 ± 0.34	0.54 ± 0.33	0.58	380.05	5	PACA 78^***^	
S5	Štefánikova chata	Tatry	subalpine	1709	14	33	2.29	0.39 ± 0.43	0.41 ± 0.29	0.49	167.63	1	PV 279^**^, PACA 78^***^, PRIV 7^**^, PV 4767^*^, PV 23741^*^	✓
S9	Zamkovského chata	Tatry	subalpine	1470	7	34	2.66	0.54 ± 0.34	0.49 ± 0.28	0.57	314.54	1		
S11	Kondraczka	Tatry	subalpine	1950	5	43	3.53	0.5	0.51	0.65	227.41	1		
S12	Tristar	Tatry	subalpine	1450	5	34	2.82	0.43	0.44	0.62	195.04	0		

^a^Only loci with significant deviation from HWE are listed; P-values were estimated by 1000 permutations (^*^P < 0.05, ^**^P < 0.01, ^***^P < 0.001).

**Table 2:** Genetic diversity and differentiation of foothill and subalpine populations within the target mountain ranges.

Group	No. of ind./pop.	No. of alleles	No. of private alleles	Allelic richness	H_E_	H_S_	DW index	F_ST_	Among populations variation (%)	Pairwise F_ST_ among populations (min-max values)	Among groups variation (%)
**Jeseníky Mts**											
Subalpine populations	29/2	44	0	2.61 ± 0.005	0.47 ± 0.01	0.53 ± 0.015	83.61 ± 1.17	0.03	2.76^*^	0.035	
Foothill populations	31/2	53	3	2.66 ± 0.055	0.45 ± 0.01	0.44 ± 0.01	181.36 ± 59.41	0.08	7.82^***^	0.057	
Subalpine × foothills											6.11 (p = 0.33)
**Krkonoše Mts**											
Subalpine populations	40/2	56	5	2.57 ± 0.05	0.45 ± 0.005	0.53 ± 0.005	186.06 ± 41	0.21	21.42^***^	0.132	
Foothill populations	39/4	60	3	2.69 ± 0.093	0.46 ± 0.019	0.50 ± 0.035	137.28 ± 26.41	0.05	5.47^***^	(0.035) 0.059 (0.08)	
Subalpine × foothills											4.26 (p = 0.14)
**Tatry Mts**											
Subalpine populations	31/4	68	3	2.83 ± 0.352	0.46 ± 0.038	0.58 ± 0.053	226.16 ± 44.82	0.24	24.18^***^	(0.065) 0.125 (0.174)	
Foothill populations	32/2	87	14	3.53 ± 0.195	0.58 ± 0.04	0.6 ± 0.015	395.92 ± 15.87	0.03	3.03 (p = 0.1)	0.06	
Subalpine × foothills											2.32 (p = 0.25)
Significance of differences between groups of foothill and subalpine populations				F_1,12_ = 0.09	F_1,12_ = 0.09	F_1,12_ = 0.3	F_1,12_ = 0.08				

P-values were estimated by 1000 permutations (^*^P < 0.05, ^**^P < 0.01, ^***^P < 0.001).

Additionally, we calculated for subalpine pop. S5 from the Nízké Tatry Mts and foothill pop. F5 and F8 among population variation (subalpine × foothill pop.) = 23.82% (p = 0.33), F_ST_ = 0.27, and pairwise F_ST_ among populations = (0.06) 0.11 (0.137).

Among-population differentiation (pairwise F_ST_) was low, but varied considerably from 0.04 to 0.28, with an average of 0.13 (Supplementary Table S3). In the Krkonoše and the Tatry, we observed lower F_ST_ values between foothill populations (average of 0.06 for six comparisons in the Krkonoše, and 0.06 for one comparison in the Tatry) compared to those between subalpine populations (on average 0.14 for six comparisons in the Tatry and 0.13 for one comparison in the Krkonoše). In contrast, two subalpine populations in the Jeseníky, that occupied different parts of one glacial cirque, were less differentiated from each other (0.04) than were their two foothill counterparts (0.06). Between the foothill and subalpine populations, we observed at most a moderate differentiation, with the highest mean pairwise F_ST_ values in the Krkonoše (0.10) followed by the Tatry (0.09), and the lowest values in the Jeseníky (0.08).

The results of an AMOVA (analysis of molecular variance) confirmed high intrapopulation variability by assigning the highest proportion of variation always among individuals within populations (from 97.24% to 73.27%). In congruence with pairwise F_ST_ values, two subalpine populations in the Krkonoše and four in the Tatry were more differentiated from each other than their foothill counterparts: 21.42% and 24.18% of among-subalpine population variation compared to 5.47% and 3.03% of among-foothill population variation in the Krkonoše (four populations) and the Tatry (two populations), respectively. The lowest variation (2.76%), although still significant, was found between subalpine populations from the Jeseníky. Finally, hierarchical AMOVAs within each mountain range showed that the foothill-subalpine differentiation was non-significant and accounted for little variation (from 2.32% to 6.11%, Table 2). To conclude, even though the structure results revealed some differentiation between foothill and subalpine populations, this differentiation is still markedly lower than differentiation observed between subalpine populations in two of the three mountain ranges.

### Gene flow, migration rates and model selections

**Figure 3:**
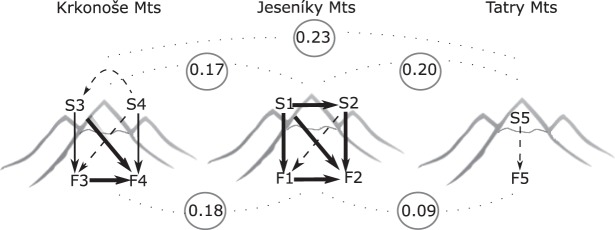
Migration and differentiation of foothill (F) and subalpine (S) populations of *P*. *elatior* in the three mountain ranges of central Europe. The values between mountain ranges are overall mean pairwise F_ST_ between foothill and subalpine populations. The arrows indicate preferred migration scenario, their width reflects estimated number of immigrants per generation (*Nm*): dashed arrow (5–21), standard arrow (68–73), and thick arrow (161–551). Created by V. Konečná.

We further addressed the role of migration in foothill-subalpine population differentiation by testing for the presence of gene flow and estimating the strength of gene flow between populations in a coalescent framework using migrate-n. Within each region, we analysed pairs of representatively sampled (≥10 individuals per population) subalpine-subalpine, foothill-subalpine, and foothill-foothill populations; for each pair, we modelled past evolutionary history under Bayesian inference of five migration models differing in presence and directionality of migration. The models with unidirectional migration gained the best support in all population pairs analysed (Table 3, Fig. 3, Supplementary Table S4). In the case of the foothill-subalpine pairs, models of unidirectional migration from subalpine to foothill populations were consistently the best models fit across different population pairs from different mountain ranges. The estimated number of immigrants (*Nm*) was overall high (>1) (ranging from 5 to 551 with an average of 160), which suggests that migration via ongoing gene flow has probably a greater effect on the extent of differentiation between populations than genetic drift (*Nm* values >1)^32^. However, besides migration, the large estimates for the number of immigrants could also at least partly reflect the recent shared ancestry of populations^33^.

We observed the lowest estimated migration rates between subalpine populations in the Krkonoše (*Nm* = 5), where subalpine populations are separated by a mountain ridge. In contrast, in the Jeseníky, a relatively weak barrier to gene flow appears to exist between the two populations residing within one glacial cirque based on the high migration rate estimated (*Nm* = 266). The highest number of immigrants in the entire dataset was also found between two foothill populations (Krkonoše, F3 and F4; *Nm* = 551) (Table 3, Fig. 3).

**Table 3:** Summary of the best fitting migration models (based on Bayes factor, see Supplementary Table S4) for foothill and subalpine populations of *P*. *elatior*, their differentiation, and numbers of immigrants per generation (*Nm*) between populations estimated by migrate-n.

Range	Model	*Nm*	Pairwise F_ST_
Jeseníky	F1 → F2	206	0.06
Krkonoše	F3 → F4	551	0.05
Jeseníky	S1 → F1	261	0.08
Jeseníky	S2 → F1	21	0.09
Jeseníky	S1 → F2	161	0.07
Jeseníky	S2 → F2	173	0.07
Krkonoše	S3 → F3	68	0.07
Krkonoše	S4 → F3	17	0.11
Krkonoše	S3 → F4	257	0.09
Krkonoše	S4 → F4	73	0.13
Nízké Tatry	S5 → F5	14	0.13
Jeseníky	S1 → S2	266	0.04
Krkonoše	S4 → S3	5	0.13

F = foothill populations, S = subalpine populations.

## Discussion

Our population genetic investigation of central European *Primula elatior* populations provided strong evidence for parallel colonization of subalpine habitats within each of the three mountain ranges that are the focus of our study (Fig. 1). In each case, subalpine populations are more closely related to the geographically closest foothill populations from the same mountain range than to their subalpine counterparts from other mountain ranges. Although island-like distributed subalpine environments are likely to trigger the evolution of recurrently adapted populations, the parallel colonization of subalpine habitats across different mountain ranges in one species has only rarely been documented with genetic data^12–14^. Future studies on parallel ecotype evolution should also focus on mechanisms behind the genetic parallelism. For instance, whether repeated selection from the standing genetic variation has important role in formation of parallel ecotypes^14,34^, whether the selection of independent mutations takes place in parallel evolution^13^, or if adaptive introgression via borrowed alleles from adapted species can facilitate this process^35^.

The example of the parallel origin of ecotypes along an elevational gradient, although with the opposite direction of colonization than in our study, was recently documented for *Heliosperma pusillum*, where distinct isolated ecotypes occupying cave entrances in foothills originated from a widespread alpine lineage^36^. The impact of elevational difference on divergence between foothill (montane) and alpine populations has been documented in *Dianthus callizonus*^37^, *Primula merrilliana*^23^, and *Solidago virgaurea*^38^. The parallel origin of ecotypes has been also documented previously with genetic data in different environments such as sand dune vs. non-dune in *Helianthus petiolaris*^39^, sand dune vs. rock in *Senecio lautus*^40^, serpentine vs. non-serpentine soils in *Solidago virgaurea*^41^, wave vs. crab predation in the mollusc *Littorina saxatilis*^42^, and freshwater vs. saltwater in the fish *Gasterosteus aculeatus*^34^. The absence of similar studies in subalpine plants might simply reflect less attention to this phenomenon of differentiation in subalpine environment.

In contrast to differentiation among the three distinct mountain ranges (mean pairwise F_ST_ = 0.13), foothill and subalpine populations within each region were less differentiated (pairwise F_ST_ ranging from 0.08 to 0.10) and accounted for a negligible and non-significant proportion of variation in AMOVA tests (from 2.32% to 6.11% variation). The variation in these values across mountain ranges likely reflects different spatial and elevational distances between subalpine and foothill populations in each region as well as heterogeneity of habitats. In the Jeseníky and northern part of the Tatry (S9, S11; Vysoké/Belanské Tatry) we observed a nearly continuous occurrence of *P*. *elatior* along an elevational gradient (corresponding with mean pairwise F_ST_ between foothill and subalpine populations of 0.08 and 0.07, respectively)^43,44^. In contrast, in the Krkonoše and the Nízké Tatry, there was a gap in the occurrence of several hundred meters, which can restrict gene flow (reflected by higher mean pairwise F_ST_ of 0.10 and 0.13, respectively), as was also revealed in *Knautia* in the Krkonoše^45^. Available population genetic studies have documented that the levels of differentiation among foothill and alpine/subalpine populations may vary considerably among species, from high (mean pairwise F_ST_ = 0.19 in *Primula merrilliana*^23^) to low (mean pairwise F_ST_ = 0.02 in *Arabidopsis arenosa*^46^ also from the Tatry).

Our study shows that genetic differentiation along an elevational gradient may vary within a species, from one mountain range to another, although this pattern appears to be less extreme in *Primula* relative to *Heliosperma*, where the individual alpine-foothill pairs varied considerably in differentiation (mean F_ST_ 0.17–0.43)^36^. The extent of differentiation likely indicates if populations have an older or younger origin/divergence. Besides elevational differences, lower or comparable differentiation to our study was also observed in two *Primula* species between grassland and forest habitats in lowland habitats at a fine-scale of several kilometres (mean F_ST_ = 0.02 in *P*. *elatior*^16^ and 0.08 in *P*. *veris*)^47^. Our observation in *P*. *elatior* thus suggests that elevation is generally a weak isolating barrier whose strength may further vary across independent colonization events in different mountains.

The relatively low foothill-subalpine differentiation that we observed in *P*. *elatior* is likely due to high levels of retained ancestral polymorphism and/or persisting gene flow along the elevational gradient. This was suggested by coalescent modelling in which we found consistent support for models with unidirectional migration from subalpine to foothill populations. The unidirectional migration likely reflected gene flow after the colonization of subalpine habitats in postglacial times. This was the best supported model in all three mountain ranges, however, with varying estimated strength across the regions. Such unidirectional downslope migration has been observed also in *P*. *merrilliana*^23^, in which the seed mobility is generally restricted in a similar way to *P*. *elatior* due to the lack of any long-distance seed dispersal adaptation^48^. In the mountains, seed flow may be further enhanced by washing downslope by mountain streams, but pollen flow may be restricted along an elevational gradient due to differences in flowering phenology between populations at different elevations^4^. In the closely related species *P*. *veris*, differences in the flowering phenology affect gene flow even among distinct habitats in the same (lowland) elevation^47^.

In contrast to the low foothill-subalpine differentiation that we observed, individual subalpine populations were the most highly differentiated entities within two of the three studied regions (21.42% and 24.18% variation in the Krkonoše and the Tatry, respectively, split into distinct genetic clusters, Fig. 2). For generalization of the differentiation of subalpine populations, however, more populations shall be compared. High differentiation between subalpine populations in the Krkonoše has been documented also in another species growing in the glacial cirques – *Gentiana pannonica* (14.15%)^49^. The Jeseníky was the only exception, in which both subalpine populations were restricted to a single glacial cirque with likely high opportunities for gene flow and this is likely responsible for the low among-population differentiation and F_ST_ values. These results suggest that mountain ridges may act as relatively strong migration barriers, which can affect gene flow more strongly than elevational differences between subalpine and foothill populations. Although higher differentiation among subalpine populations may also reflect past bottleneck events during their origin from their foothill counterparts, we do not consider this likely. We have not observed significantly reduced levels of diversity in these populations (Table 2) – a clear sign of past bottleneck in populations^50^.

In contrast to the differentiation observed among subalpine populations, differentiation among foothill populations was low (3.03–7.82% in different regions). This relatively low differentiation is may be the product of a higher density and large population size of foothill populations. Pollination is more efficient in large populations and the larger population size may provide more opportunities for gene flow among populations^16,51^.

Our results show that subalpine colonization did not appear to lead to a loss of genetic diversity relative to foothill populations. The differences in genetic diversity of subalpine vs. foothill populations were non-significant over the replicated mountain ranges (Table 2). Moreover, neither index of genetic diversity shows any consistent trend across the regions with respect to the elevational groups (Supplementary Fig. S2). On the other hand, foothill populations from the Tatry were together the most genetically diverse of all populations included in our study. This could be a consequence of the foothill habitat^16,17,52^, which may have experienced the long-term isolation of populations in this area serving as a glacial refugium for temperate species^46,53^. Generally, subalpine populations of *P*. *elatior* appear to be able to maintain genetic diversity equal to foothill populations despite smaller population sizes and the spatial isolation of the glacial cirques in which they occur. This could be due to a large size of the initial colonizing population (ruling out founder effect) and the relative stability of the habitat in postglacial time providing good conditions for the persistence of sufficiently large populations. An additional non-exclusive explanation might be a gradual and relatively slow pace of the shifts of the treeline during the Holocene, which might have maintained sufficient population sizes without bottlenecks, and thus preserve genetic diversity during the colonization process^23^.

In summary, we describe a case of parallel colonization of subalpine habitats from multiple foothill gene pools. Our results imply that there is a distinct mountain diversity in the subalpine habitats – the subalpine populations are genetically differentiated from their foothill counterparts and from each other. In addition, subalpine populations regularly preserve genetic diversity at similar levels relative to their foothill counterparts. However, ongoing gene flow, in particular from subalpine to foothill habitats together with low levels of differentiation, likely linked to a recent (postglacial) origin of the subalpine populations, seems to prevent any stronger differentiation along the elevational gradient that may lead to speciation.

## Methods

### Study species and sampling

*Primula elatior* is an outcrossing perennial plant with a basal rosette of leaves. Flowers are produced in an umbel, characterized by distyly and self-incompatibility^25^. The main pollinators are Hymenoptera (mostly bumblebees) and Diptera^54,55^. Seed mobility in this species is generally restricted due to lack of any adaptations for long-distance dispersal, for instance, compared to closely related species *P*. *vulgaris*^48^. Therefore, seeds are dispersed autonomously for short distances, and occasionally they can be washed downslope from streamside habitats.

Plant material was collected in 2015–2016 in the three mountain ranges in central Europe: the Jeseníky, the Krkonoše, and the Tatry. In the Jeseníky and the Krkonoše, we sampled all known populations in subalpine glacial cirques and representative set from distinct valleys in the foothills. In the Tatry, we included populations from two distinct subalpine parts: the southern mountain range of the Nízké Tatry, the northern-eastern mountain range of the Vysoké/Belanské Tatry, and representative foothill populations in the basins between these two mountain ranges (called overall the Tatry). Our dataset comprises in total 202 individuals from 16 populations (Table 1 and Supplementary Table S1). In each mountain range, we sampled multiple foothill (below treeline) and subalpine (above treeline) populations. Individuals in the populations were sampled randomly, but with a minimum distance of approximately 1 m between individuals to avoid collecting clones. Leaf tissue from five to twenty individuals per population was immediately dried in silica gel for subsequent DNA extraction and microsatellite genotyping.

### Microsatellite genotyping

Genomic DNA was extracted from dry tissue using a modified NaCl/CTAB protocol^56^. We employed 12 microsatellite loci developed by Van Geert *et al*.^57^, Bickler *et al*.^58^, and Seino *et al*.^59^ (Supplementary Table S2). From those, seven loci, originally developed for *P*. *veris*^58^, were successfully cross-amplified in *P*. *elatior*: PV 23741, PV 21795, PV 27775, PV 4767, PV 23424, PV 19773, and PV 279. The others (Paca 11, Paca 38, Paca 78, Priv 4, and Priv 7) have been already cross-amplified in *P*. *elatior* by Seino *et al*.^59^ and Van Geert *et al*.^57^. Our cross-amplification of the 12 loci was successful, meaning that we consistently amplified variable loci across the sample set, contrary to three additional loci Paca 404^59^, PV 8880^58^, and PV 26720^58^, which were not consistently amplified, and therefore not employed in the study. The fluorescently labelled primers (dyes: PET, NED, 6-FAM, and VIC; Applied Biosystems) were designed into two multiplexes based on the results from a complementary threshold analysis in Multiplex manager 1.2. Microsatellite loci were amplified using the QIAGEN Multiplex PCR Kit, with a total reaction volume of 5 μl of QIAGEN mix. The mix contained 0.25 μl of forward primer and 0.25 μl of reverse primer, 1 μl of ddH_2_O, 2.5 μl of Master Mix, and we added 1 μl (10 ng) of DNA. The PCR amplification was conducted in a thermocycler (Eppendorf Mastercycler Pro) under the following conditions for both multiplexes: 5 min of denaturation at 95 °C, followed by 35 cycles of 95 °C at 30 s, 57 °C for 90 s, 72 °C for 40 s, and a final extension of 68 °C for 30 min. Amplification products were separated using 3130xl Genetic Analyser (DNA laboratory of Faculty of Science, Charles University, Prague) with GeneScan 500 LIZ (Applied Biosystems) as an internal standard.

### Genetic structure and diversity

Allele sizes were determined in genemarker 2.6 (SoftGenetics). We checked possible presence of null alleles, stuttering, large allele dropout by the program micro-checker 2.2.3^60^. None of the loci showed presence of null alleles in more than half of the populations (maximum seven for Priv 4 and six for Paca 78), and we thus retained all 12 microsatellite loci in analyses. We tested if loci significantly deviated from HWE in each population in R, package pegas^61^. None of the populations had significant deviation from HWE in more than half of the loci (maximum seven in S5) (Table 1).

First, we explored population structure in the entire dataset as well as separately for each region using Bayesian clustering in structure 2.3.3^62^ employing Abel HPC cluster of the University of Oslo. We used independent allele frequencies model with admixture, which allows for mixed ancestry of individuals. The number of clusters was set from K = 1 to K = 10 for entire dataset and from K = 1 to K = max, in which “max” equalled number of sampled populations in particular regions (K = 6 for each of the Krkonoše and the Tatry datasets, K = 4 for the Jeseníky dataset). Analysis for each K was performed with 20 replicates, the initial length of burn-in period 100,000 and 1,000,000 of Markov chain Monte Carlo (MCMC) replicates after burn-in. Similarity coefficients among runs of the same K^63^ were calculated using Structure-sum-2009 script^31^ in R 3.3.2^64^. For an optimal number of clusters (K), we considered the partition, where the rising likelihood of K values started to flatten and which also exhibited high similarity among replicated runs for that particular K (Supplementary Fig. S1). Some analyses allowed several possibilities for the optimal number of K, due to the hierarchical genetic structure of populations^65^, in that case, we presented several partitions. Outputs from structure analyses were graphically visualized in Structure Plot V_2.0_^66^.

Further, we visualized relationships among individuals and populations using distance-based approaches. Firstly, genetic relationships among individuals were plotted in centred principal component analysis (PCA) calculated in R, package adegenet^67^. Secondly, networks of pairwise F_ST_ distance among populations were created based on neighbor-net algorithm in SplitsTree 4.13.1^68^. Nei’s pairwise F_ST_s^69^, in which heterozygosities are weighted by group sizes, and therefore comparison between populations with different sizes of individuals is possible, were calculated in R, packages adegenet^67^ and hierfstat^70^.

In order to test differences in population genetic properties, we calculated descriptive statistics of all populations with respect to small number of individuals and imbalance sampling. Due to varying number of individuals samples per populations (from five to 20 individuals) we employed following subsampling strategy to rule out the effect of varying sample size: five individuals per population were randomly selected, we calculated the corresponding statistics and repeated the process 100 times and we further presented mean value with standard deviation from 100 replicates (https://github.com/MarekLipan/Population-subsampling/blob/master/Genind_subsampling_func.R). We calculated observed heterozygosity (H_O_) and expected heterozygosity (H_E_) by subsampling in R, packages adegenet^67^ and hierfstat^70^. Further, we calculated the numbers of alleles, allelic richness (with reference population size of five individuals per population, 1,000 permutations), and Nei’s unbiased estimator for gene diversity (H_S_)^71^, which is corrected for small sample size, in Microsatellite analyser (MSA) 4.05^72^. Furthermore, we quantified the proportion of rare alleles using the frequency of down-weighted marker index (DW index), calculated as a ratio of means from the presence-absence matrix of alleles, which makes the measure less sensitive to different number of individuals per population^31^. DW index was calculated using R-script AFLPdat^31^. Finally, a number of private alleles was counted in R, package PopGenKit^73^. Differences in allelic richness, H_E_, H_S,_ and in DW index between foothill and subalpine group were tested by hierarchical ANOVA with region as a random effect factor in R, package stats. Further, hierarchical structuring of genetic variation among populations within foothill vs. subalpine group in each region was revealed by analysis of molecular variance (AMOVA) in Arlequin 3.1^74^. AMOVAs were calculated by the method of the number of different alleles (F_ST_-like) with 1,000 permutations. Finally, the structuring of variation among foothill and subalpine populations in each region was also explored by hierarchical AMOVAs.

### Estimation of gene flow direction and migration rates

To identify the direction and intensity of migration among the foothill and subalpine populations, we searched for optimal models of migrations between populations within each target mountain range in a coalescent framework using migrate-n version 3.6.11^75^. We have chosen two pairs of foothill and subalpine populations from the Jeseníky, the Krkonoše, and one pair from sub-range of the Tatry (Nízké Tatry), focusing on populations with the maximum number of genotyped individuals (n ≥ 10). To keep the simulation scenarios feasible, we worked with two-population models that were iterated among all possible population combinations within each mountain range: we analysed pairs of subalpine-subalpine, foothill-subalpine, and foothill-foothill populations. For each pair, we modelled past evolutionary history under Bayesian inference of five migration models differing in presence and directionality of migration. We allowed bidirectional migration between two populations (model 1), unidirectional migration (models 2 and 3), panmixia (model 4 assuming that two populations belong to one panmictic population), and zero migration between two separate populations (model 5). In case of foothill-subalpine comparisons, the model 2 allowed migration from foothill to subalpine while the model 3 assumed only migration from subalpine to foothill populations.

We used microsatellite data type with Brownian motion microsatellite model. The mutation rate was set constant over all loci. migrate-n estimated two parameters Theta - *θ* (mutation scaled population size) and *M* (mutation scaled immigration rate). The starting values of *θ* and *M* were calculated from Wright’s F_ST_ using prior values (for *θ*: minimum = 0.004, delta ranged from 0.9 to 2.0, maximum ranged from 9.0 to 20, bins = 500; for *M*: minimum = 0, delta ranged from 80 to 100, maximum ranged from 800 to 1000, bins = 500). Prior distributions of population sizes and migration rates were set based on the personal knowledge of the populations from the field with a broader range of both parameters with the aim to achieve the best searching of the space. To reach the stable states we ran each model for each pair multiple times (at least five). We have also checked the effective sample size, which was well over 500 (approximately 3000–5000). After burn-in of 20,000, we sampled 500,000 states from a single Markov chain, one every 5,000 steps. Four chains were run in parallel with heating static scheme (temperatures: 1.0, 1.5, 3.0, 10,000). According to Hodel *et al*.^32^, based on *θ* and *M* parameters we calculated a number of immigrants per generation following the formula: *Nm* = [(*θ*_*x*_ ∗ *M*_*y→x*_)/4] (for nuclear loci). For calculating *Nm* values, we used median values for both *θ* and *M* parameters.

To select the most likely model among the five models for each population pair, we used Bayes factors comparison. Bayes factor allows comparing nested and non-nested models, without assuming normality, or large samples^76,77^. We calculated natural log Bayes factors following the formula: *LBF* = ln [*mL* (model_1_)] − ln [*mL* (model_2_)], in which model_1_ is a model with the highest marginal likelihood and model_2_ is each of the other models. We used “Bezier” approximated marginal likelihood calculated using the thermodynamic integration with the heating scheme described above). Marginal likelihood is the integral of the likelihood function over the complete parameter range. Afterwards, we calculated the probability of each model following the formula:

$$Pro{b}_{mode{l}_{i}}=\frac{mLmode{l}_{i}}{{\sum }_{j}^{n}\,mLmode{l}_{j}},$$

in which *mLmodel*_*i*_ is the marginal likelihood of *model*_*i*_ and $\sum _{j}^{n}\,mLmode{l}_{j}$ is the sum of the marginal likelihoods of all other models.

## Supplementary information

Supplementary information file

## Data Availability

All data generated or analysed during this study are included in this article (and its Supplementary Information Files).
