# Supplementary material for: Parallel colonization of subalpine habitats in the central European mountains by Primula elatior
Source: Sci Rep. 2019 Mar 1;9:3294. doi: 10.1038/s41598-019-39669-2 (PMC6397301; doi:10.1038/s41598-019-39669-2)
Supplement: Supplementary file 1 — Supplementary information file [file 41598_2019_39669_MOESM1_ESM.pdf]

Supplementary Information file

Parallel colonization of subalpine habitats in the central European mountains by *Primula elatior*

Veronika Konečná[1,2]*, Michael D. Nowak[3], Filip Kolář[1,2,4]

[1]*Department of Botany, Faculty of Science, Charles University, Benátská 2, CZ-128 00 Prague, Czech Republic*

[2]*Institute of Botany, The Czech Academy of Sciences, Zámek 1, CZ-252 43 Průhonice, Czech Republic*

[3]*Natural History Museum, University of Oslo, Sars' gate 1, NO-0562 Oslo, Norway*

[4]*Department of Botany, University of Innsbruck, Sternwartestraße 15, AT-6020 Innsbruck, Austria*

**Correspondence to Veronika Konečná (konecnv@natur.cuni.cz)*

Supplementary Table S1. Locality details on 16 populations of *Primula elatior* sampled in the study.

Pop. ID	Locality	Locality details
F1	Žďárský potok u Rýmařova	CZ, Olomoucký kraj, Žďárský potok u Rýmařova, valley meadow along the river Slatinný potok, 735 m, 49.9679331N, 17.208975E, V. Konečná
F2	V Mlýnkách	CZ, Olomoucký kraj, meadow in the forest near Javořický potok, 4 km SW from Jeseník town, 618 m, 50.198507N, 17.15203E, V. Konečná
S1	Velká Kotlina	CZ, Olomoucký kraj, upper part of the Velká Kotlina glacial cirque in grassy slope, 1397 m, 50.0566111N, 17.2334444E, V. Konečná
S2	Velká Kotlina - edge	CZ, Olomoucký kraj, Velká Kotlina - edge of the glacial cirque above treeline, 1301 m, 50.058142N, 17.23931E, V. Konečná
F6	Michlův mlýn	CZ, Královéhradecký kraj, slope above the road along forest edge, 3 km S from Špindlerův Mlýn, 658 m , 50.6989081N, 15.58808E, V. Konečná
F7	Svatý Petr	CZ, Královéhradecký kraj, meadow above the road in Svatý Petr, 816 m , 50.7251831N, 15.6313581E, V. Konečná
F3	Strážné	CZ, Královéhradecký kraj, meadow in Strážné, 3 km NE from Vrchlabí, 776 m, 50.6619181N, 15.613365E, V. Konečná
F4	Spálený mlýn	CZ, Královéhradecký kraj, river bank in the valley of Malá Úpa river, 5 km NE from Pec pod Sněžkou, 788 m, 50.7067281N, 15.8045919E, V. Konečná
S3	Velká Kotelní jáma	CZ, Královéhradecký kraj, upper part of the Velká Kotelní jáma glacial cirque along the stream, limestone, 1280 m, 50.7516589N, 15.534064E, V. Konečná, L. Harčariková
S4	Malá Sněžná jáma	PL, Województwo dolnośląskie, basalt outcrop in the NW part of the Malá Sněžná jáma glacial cirque, 1368 m, 50.7833086N, 15.5550314E, J. Harčarik, V. Konečná
F8	Ždiar, Tatranská Kotlina	SK, Prešovský kraj, meadow in the valley Tatranská Kotlina of the river Biela, 6 km SE from Ždiar, 769 m, 49.23075N, 20.318285E, V. Konečná
S9	Zamkovského chata	SK, Prešovský kraj, river bank of the stream Malý Studený potok in Malá Studená dolina valley, 1470 m, 49.173491N, 20.219337E, V. Konečná
S11	Kondraczka	PL, Województwo małopolskie, snowbed on the NE slope of Kopa Kondraczka mountain, 1950 m, 49.236847N, 19.931016E, F. Kolář
S12	Tristar	SK, Prešovský kraj, grassy slope in Tristarska dolina, 3 km SW from Ždiar, 1450 m, 49.252393N, 20.207481E, F. Kolář
F5	Poludnica	SK, Žilinský kraj, along the forest edge of meadow in Janská dolina valley in Liptovský Ján, 1055 m, 49.026427N, 19.652469E, V. Konečná
S5	Štefánikova chata	SK, Žilinský kraj, meadow below the Štefániková chata cottage at the top of dolina Štiavnica valley, 1709 m, 48.9265242N, 19.6494031E, V. Konečná

Supplementary Table S2. Summary of 12 microsatellite loci.

Locus name	Genbank accession number	Dye	Primer sequences	Repeat sequence	Size range (bp)	N alleles	Multiplex
Paca 11	KJ134992	6-FAM	F: TTCGTGATGAAGTTGACTTTTATG<br>R: AAACAGCAATATCAGAGTCCAGA	$(TC)_{12}$	83 - 101	9	1
Paca 38	KJ134995	NED	F: ACATTCAGCGAGTGATTTGG<br>R: GCAGAGCGTGGTCTATGTT	$(AAC)_{5}$	189 - 210	6	2
Paca 78	KJ135002	VIC	F: TGTGCGACTGCCTCTATCTC<br>R: GACTGAGAAGACATATGTTGAAAGA	$(CT)_{11}$	116 - 164	22	1
PV 279	KF220372	NED	F: GTCCACCACCCTCTTATCCG<br>R: CCTCGAGTTGGAGTACTTGC	$(TG)_{17}$	101 - 147	18	1
PV 23424	KF220365	PET	F: GCAGTGGATGGGTATGAAAG<br>R: GTGGTAGCTTCTTGTTCAGGG	$(CAA)_{7}$	159 - 183	9	1
PV 23741	KF220368	PET	F: GCTTGAACGGTGATAGCGAC<br>R: GTATCGGTCGTGCCATTTCG	$(TGT)_{7}$	158 - 179	8	2
PV 21795	KF220366	VIC	F: TCCTACATCGCAACATCTTCTG<br>R: CCACATACGGCTCTGACATC	$(ATAC)_{7}$	169 - 225	12	1
PV 4767	KF220370	NED	: TGGTATTTCTTCTGCTTCTTTGC<br>R: ACGGCTAAAGTACCACCACC	$(CTT)_{10}$	104 - 140	12	2
PV 27775	KF220373	VIC	F: TCCGAGTACAGATCATGGAAC<br>R: CCTGCATGTACTAGAGCCAAC	$(CATA)_{13}$	163 - 215	13	2
PV 19773	KF220375	6-FAM	F: TTCAATTTCTGTGAAGGCTGG<br>R: TCGGGATATGCCAATCAATGC	$(GTT)_{13}$	191 - 221	10	2
PRIV 4	DQ858205	PET	F: ACTCTTCTTGTTCCTCTCCCA<br>R: ACCTAGTTCCTCGCTCCACA	$(GT)_{24}$	75 - 123	25	1
PRIV 7	DQ858207	6-FAM	F: GATTCCAACAACTACGGTTCA<br>R: CAATGAAAATCTACATGTTACG	$(GT)_{14}$	190 - 252	28	1

Supplementary Table S3. Pairwise F_{ST} among populations in the three target mountain ranges; F = foothill populations, S = subalpine populations.

**Pop. ID**	**Krkonoše Mts**						**Jeseníky Mts**				**Tatry Mts**				
	S3	S4	F4	F3	F6	F7	F1	F2	S2	S1	F5	S5	S11	S9	F8
S3															
S4	0.132														
F4	0.092	0.132													
F3	0.068	0.113	0.050												
F6	0.091	0.131	0.076	0.035											
F7	0.056	0.109	0.080	0.046	0.068										
F1	0.214	0.203	0.202	0.199	0.229	0.179									
F2	0.174	0.169	0.153	0.147	0.179	0.162	0.057								
S2	0.155	0.172	0.139	0.142	0.177	0.140	0.091	0.069							
S1	0.157	0.196	0.173	0.159	0.202	0.163	0.075	0.071	0.035						
F5	0.079	0.103	0.071	0.085	0.102	0.084	0.103	0.075	0.079	0.096					
S5	0.233	0.226	0.221	0.257	0.275	0.244	0.227	0.207	0.187	0.203	0.132				
S11	0.102	0.114	0.133	0.115	0.169	0.176	0.090	0.084	0.091	0.101	0.052	0.148			
S9	0.130	0.151	0.140	0.125	0.167	0.184	0.147	0.128	0.114	0.141	0.070	0.174	0.113		
F8	0.114	0.118	0.113	0.117	0.146	0.119	0.142	0.123	0.097	0.132	0.060	0.137	0.068	0.079	
S12	0.127	0.129	0.173	0.145	0.200	0.219	0.116	0.119	0.114	0.153	0.070	0.152	0.098	0.122	0.065

Supplementary Table S4. Comparisons of different migration models for foothill and subalpine populations using Bayes factor. We calculated for each model: Bezier approximated marginal likelihood (Bezier ln*mL*), delta Bezier ln*mL* (Delta), natural log Bayes factor (LBF), probability (values lower than 10^{-6} were replaced by zero), ϑ, *M* parameters, and number of immigrants (*Nm*); F = foothill populations, S = subalpine populations

Jeseníky Mts	Model	Bezier ln*mL*	Delta	LBF	Probability	**ϑ S1†**	**ϑ S1 (CI and median) ***	**ϑ S2†**	**ϑ S2 (CI and median) ***	***M* S1 → S2†**	***M* S1 → S2 (CI and median) ***	***Nm***
S1-S2	Full migration	-980.34	-166.96	166.96	0							
	S1 → S2	-813.38	0	0	1	1.34, 1.43	(0.96) 1.38 (1.61)	4.56, 4.67	(0.98) 4.63 (6.37)	196, 236.5	(97.6) 229.6 (385)	266
	S2 → S1	-855.46	-42.08	42.08	0							
	Panmictic	-867.7	-54.32	54.32	0							
	Without migration	-1575.3	-761.92	761.92	0							
						**ϑ F1**		**ϑ S1**		***M* S1 → F1**		***Nm***
F1-S1	Full migration	-1171.49	-389.15	389.15	0							
	F1 → S1	-811.64	-29.3	29.3	0							
	S1 → F1	-782.34	0	0	1	2.15, 6.04	(0.72) 5.24 (10.5)	2.33, 2.37	(1.62) 2.39 (3.21)	169, 228	(126) 199 (430)	261
	Panmictic	-816.81	-34.47	34.47	0							
	Without migration	-2313.66	-1531.32	1531.32	0							
						**ϑ F2**		**ϑ S1**		***M* S1 → F2**		***Nm***
F2-S1	Full migration	-837.58	-190.01	190.01	0							
	F2 → S1	-674.26	-26.69	26.69	0							
	S1 → F2	-647.57	0	0	1	3.78, 4.86	(0.52) 3.9 (11.64)	1.42, 1.45	(0.84) 1.92 (2.02)	159, 185.52	(72) 165 (356)	161
	Panmictic	-722.33	-74.76	74.76	0							
	Without migration	-1590.13	-942.56	942.56	0							
						**ϑ F1**		**ϑ S2**		***M S2* → F1**		***Nm***
F1-S2	Full migration	-1092.46	-294.91	294.91	0							
	F1 → S2	-831.07	-33.52	33.52	0							

	S2 → F1	-797.55	0	0	1	2.03, 5.28	(0.5) 3.32 (9.52)	1.83, 1.69	(0.7) 1.78 (2.35)	15, 35.61	(15) 25.5 (80)	21
	Panmictic	-910.48	-112.93	112.93	0							
	Without migration	-2277.58	-1480.03	1480.03	0							
						**ϑ F2**		**ϑ S2**		***M* S2 → F2**		***Nm***
F2-S2	Full migration	-987.08	-207.17	207.17	0							
	F2 → S2	-781.36	-1.45	1.45	0.19							
	S2 → F2	-779.91	0	0	0.81	1.49, 3.9	(0.63) 3.32 (8.61)	2.06, 2.09	(1.47) 2.12 (2.64)	271, 350.77	(168) 331 (576)	173
	Panmictic	-827.65	-47.74	47.74	0							
	Without migration	-1559.47	-779.56	779.56	0							
						**ϑ F1**		**ϑ F2**		***M* F1 → F2**		***Nm***
F1-F2	Full migration	-1123.44	-369.97	369.97	0							
	F1 → F2	-753.47	0	0	1	1.62, 1.64	(1.12) 1.66 (2.08)	2.1, 2.81	(0.24) 2.38 (6.12)	281, 403	(148) 347 (558)	206
	F2 → F1	-789.13	-35.66	35.66	0							
	Panmictic	-790.41	-36.94	36.94	0							
	Without migration	-2700.27	-1946.8	1946.8	0							
**Krkonoše Mts**						**ϑ S3**		**ϑ S4**		***M* S4 → S3**		***Nm***
S3-S4	Full migration	-1830.12	-335.46	335.46	0							
	S3 → S4	-1545.92	-51.26	51.26	0							
	S4 → S3	-1494.66	0	0	1	1.52, 1.43	(0.74) 1.43 (2.16)	1.16, 1.14	(0.67) 1.16 (1.41)	11, 11.61	(0.1) 15 (28)	5
	Panmictic	-1710.94	-216.28	216.28	0							
	Without migration	-7651.4	-6156.74	6156.74	0							
						**ϑ F3**		**ϑ S3**		***M* S3 → F3**		***Nm***
F3-S3	Full migration	-1605.82	-421.45	421.45	0							

	F3 → S3	-1195.27	-10.9	10.9	0							
	S3 → F3	-1184.37	0	0	1	7.39, 6.3	(4.3) 6.61 (8.98)	2.3, 2.13	(1.64) 2.23 (2.68)	39, 43.92	(10) 41 (72)	68
	Panmictic	-1312.92	-128.55	128.55	0							
	Without migration	-10469.49	-9285.12	9285.12	0							
						***ϑ* F4**		***ϑ* S3**		***M* S3 → F4**		***Nm***
F4-S3	Full migration	-1352.33	-530.44	530.44	0							
	F4 → S3	-823.53	-1.64	1.64	0.16							
	S3 → F4	-821.89	0	0	0.84	8.24, 7.97	(1.83) 7.97 (14.25)	1.37, 1.41	(0.93) 1.43 (1.77)	115, 150.88	(22) 129 (308)	257
	Panmictic	-856.62	-34.73	34.73	0							
	Without migration	-1158.11	-336.22	336.22	0							
						***ϑ* F3**		***ϑ* S4**		***M* S4 → F3**		***Nm***
F3-S4	Full migration	-1266.68	-131.95	131.95	0							
	F3 → S4	-1168.32	-33.59	33.59	0							
	S4 → F3	-1134.73	0	0	1	2.6, 4.14	(0.57) 3.65 (9.42)	1.16, 1.15	(0.78) 1.19 (1.47)	17, 19.67	(0.2) 19 (34)	17
	Panmictic	-1178.82	-44.09	44.09	0							
	Without migration	-6630.48	-5495.75	5495.75	0							
						***ϑ* F4**		***ϑ* S4**		***M* S4 → F4**		***Nm***
F4-S4	Full migration	-1276.58	-297.26	297.26	0							
	F4 → S4	-1020.07	-40.75	40.75	0							
	S4 → F4	-979.32	0	0	1	4.45, 4.53	(0.45) 4.49 (9.5)	1.82, 1.58	(1.35) 1.73 (2.22)	57, 99.7	(57) 65 (184)	73
	Panmictic	-1073.67	-94.35	94.35	0							
	Without migration	-7146.99	-6167.67	6167.67	0							
						***ϑ* F3**		***ϑ* F4**		***M* F3 → F4**		***Nm***
F3-F4	Full migration	-825.45	-143.89	143.89	0							

	F3 → F4	-681.56	0	0	0.9998	1.97, 1.92	(1.38) 1.97 (2.43)	2.45, 6.94	(0.57) 7.04 (12.72)	325, 323.84	(96) 131 (568)	551
	F4 → F3	-690.23	-8.67	8.67	0.0002							
	Panmictic	-723.33	-41.77	41.77	0							
	Without migration	-1720.61	-1039.05	1039.05	0							
**Nízké Tatry Mts**						***ϑ* F5**		***ϑ* S5**		***M* S5 → F5**		***Nm***
F5-S5	Full migration	-1954.06	-376.57	376.57	0							
	F5 → S5	-1639.67	-62.18	62.18	0							
	S5 → F5	-1577.49	0	0	1	10.94, 11.38	(6.56) 11.22 (16.96)	1.96, 1.76	(1.2) 1.78 (2.24)	3, 3.68	(0.7) 5 (11)	14
	Panmictic	-1764.48	-186.99	186.99	0							
	Without migration	-4686.42	-3108.93	3108.93	0							

ᵻ mean and mode values; *confidence intervals (2.5 % - 97.5 %) with median values

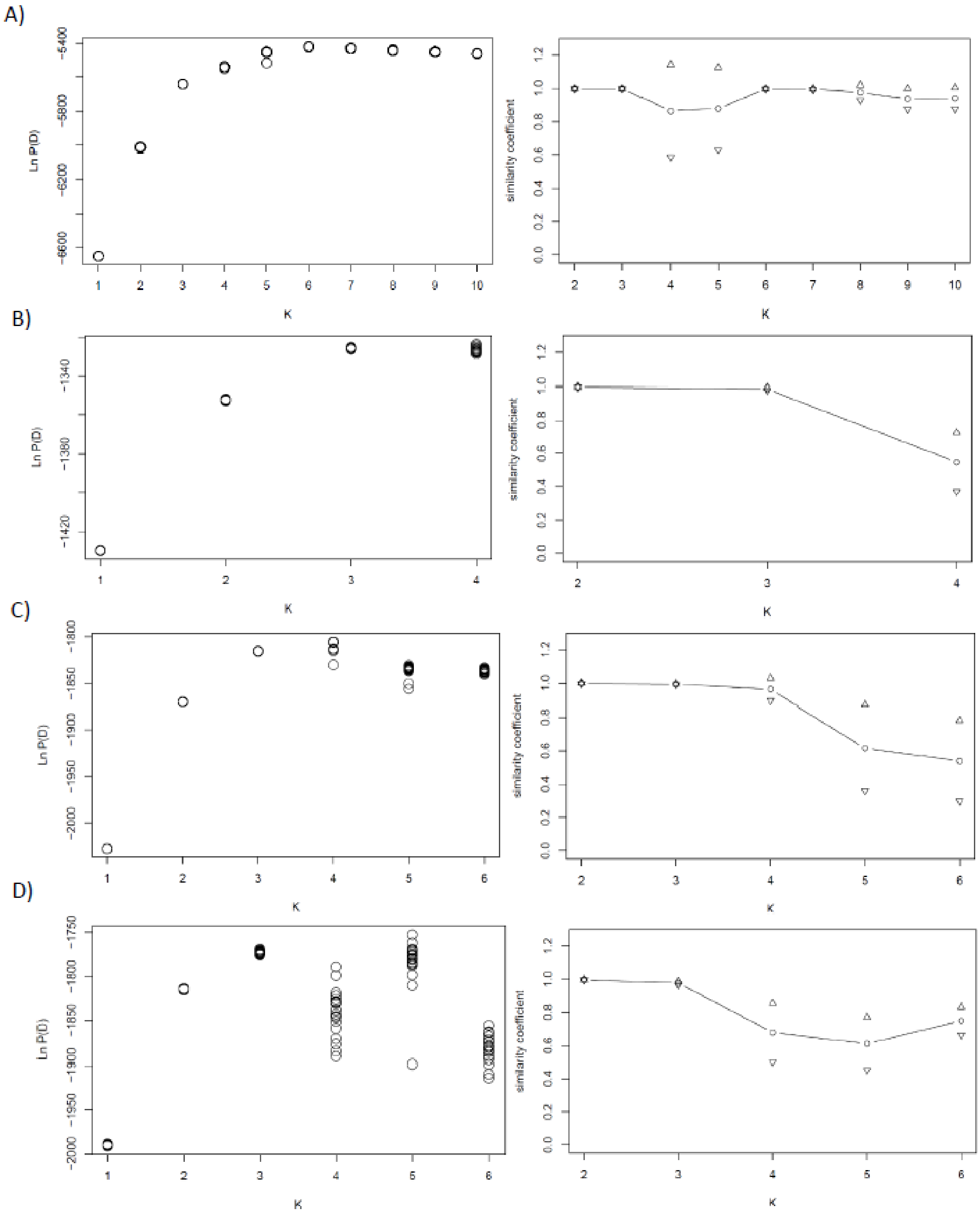

Supplementary Figure S1. Summary of STRUCTURE analyses runs in 20 replicates for each K, values of Ln probability are plotted against number of K (left column), coefficients of similarities among runs for particular K (right column). A) Complete dataset (all three target mountain ranges), B) the Jeseníky, C) the Krkonoše, and D) the Tatry

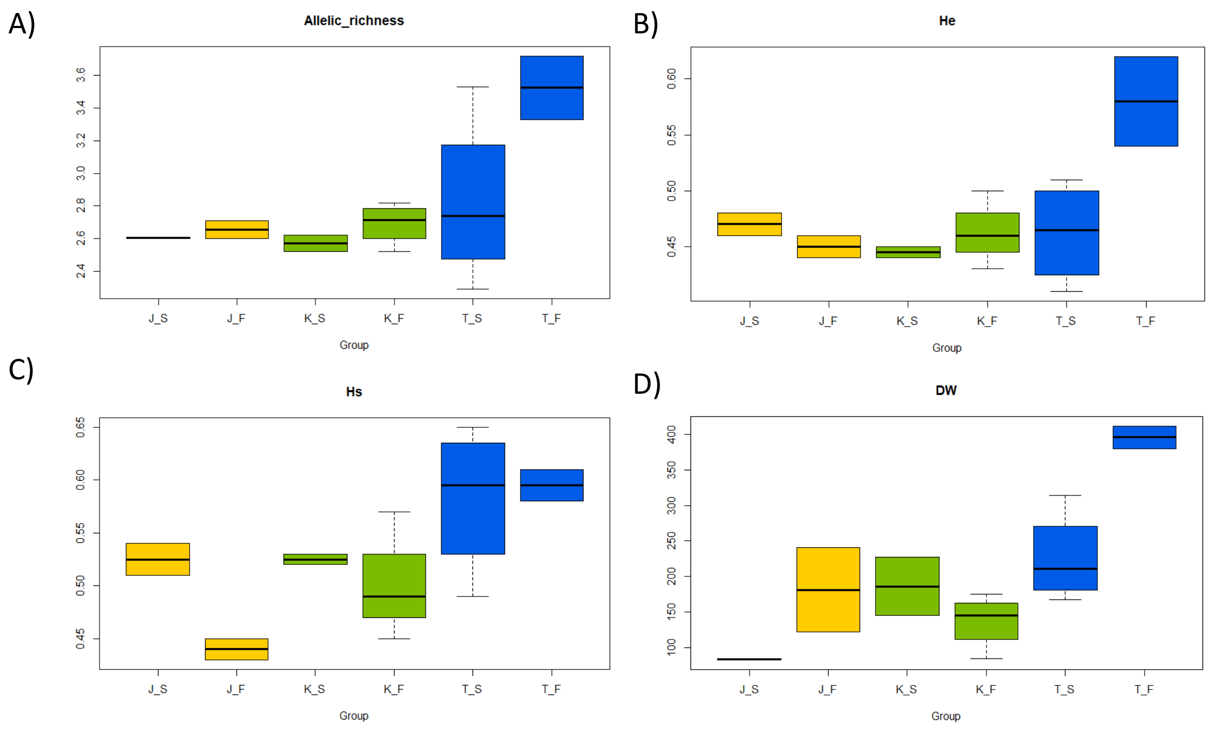

Supplementary Figure S2. Variation in genetic diversity of foothill and subalpine populations (J = the Jeseníky, K = the Krkonoše, T = the Tatry; F = foothill populations, S = subalpine populations), A) allelic richness, B) H_E (expected heterozygosity), C) H_S (gene diversity), and D) DW index.
